# Expression of hepatic miRNAs targeting porcine glucocorticoid receptor (GR) 3′UTR in the neonatal piglets under a maternal gestational betaine supplementation

**DOI:** 10.1016/j.dib.2015.11.037

**Published:** 2015-11-26

**Authors:** Demin Cai, Haoyu Liu, Mengjie Yuan, Shifeng Pan, Yimin Jia, Ruqian Zhao

**Affiliations:** a Key Laboratory of Animal Physiology & Biochemistry, Nanjing Agricultural University, Nanjing 210095, PR China; b Department of Medical Cell Biology, University of Uppsala, Uppsala SE-75123, Sweden; c College of Veterinary Medicine, Yangzhou University, Yangzhou 225009, PR China

**Keywords:** miRNAs, GR, Betaine, Neonatal liver

## Abstract

Glucocorticoid receptor (GR) has been previously demonstrated an important transcriptional factor of hepatic metabolic genes in the neonates under a maternal gestational betaine supplementation (“Gestational dietary betaine supplementation suppresses hepatic expression of lipogenic genes in neonatal piglets through epigenetic and glucocorticoid receptor-dependent mechanisms” Cai et al., 2015 [1]). Here we provide accompanying data about the expression of hepatic miRNAs targeting porcine GR 3′UTR in the neonatal piglets. Liver samples were obtained and RNA was isolated. RNA was polyadenylated by poly (A) polymerase and then dissolved and reverse transcribed using poly (T) adapter. The diluted cDNA were used in each real-time PCR assay. The sequences of all the porcine miRNAs were acquired from miRBase (http://www.mirbase.org/). miRNAs targeting GR were predicted using the PITA algorithm. Among all the predicted miRNAs, 4 miRNAs targeting GR were quantitated by real-time PCR and miRNA-124a, which has been identified to target GR 3′UTR [2], [3], was more highly expressed in betaine-exposed neonatal livers.

**Specifications table**

**Table:** 

Subject area	Biology
More specific subject area	Animal nutrition and metabolism
Type of data	Figure of miRNAs predicted to target GR, Table of miRNAs expression
How data was acquired	Quantitative PCR analysis was performed using SYBR Premix Ex Taq™ PCR Master Mix in Mastercycler^®^ep realplex PCR detection system.
Data format	Filtered and analyzed
Experimental factors	Maternal gestational betaine supplementation
Experimental features	RNA isolation and polyadenylation; real-time PCR.
Data source location	Dafeng, Jiangsu, China
Data accessibility	Data are provided in the paper

Value of the data

• miRNAs participation in post-transcription of genes could be included in other studies of fetal programming.
• The data show new way to study porcine hepatic function of glucocorticoid receptor.
• The data may be useful as comparison with human health care studies of methyl donor supplementation in the mothers’ diets.

## 1 Data, experimental design, materials and methods

### 1.1 Liver samples

Sows were divided randomly into control and betaine groups (8 per group) while sows were fed basal diet and received betaine-supplemented (3 g/kg) diet respectively throughout the pregnancy. All sows were fed three times per day at 05:00, 10:00 and 17:00 h, respectively, with free access to water. Newborn piglets were individually weighed immediately after birth and one male piglets of the mean body weight were selected per litter and sacrificed before suckling. Liver samples were collected immediately, snap-frozen in liquid nitrogen and stored at −80 °C.

### 1.2 Analyses of miRNAs targeting GR

Glucocorticoid receptor (GR) has been previously demonstrated an important transcriptional factor of hepatic metabolic genes in the neonates under a maternal gestational betaine supplementation [1]. We further the study for analyses of miRNAs targeting porcine GR.

**Fig. 1:**
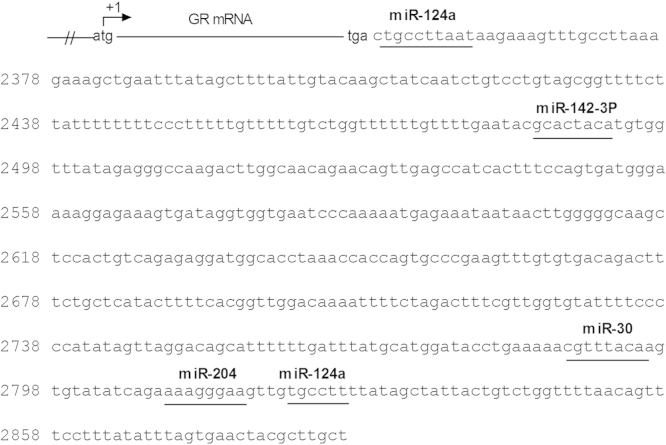
The 3′UTR of GR gene (NR3C1) were acquired from NCBI database (NM_001008481.1). The sequences of all the porcine miRNAs were acquired from miRBase (〈http://www.mirbase.org/〉). miR-124a, miR-142-3p, miR-30 and miR-204, the miRNAs were predicted to target the 3′UTR of GR with an online miRNA prediction tool [4].

The 3′UTR of GR gene (NR3C1) were acquired from NCBI database (NM_001008481.1). The sequences of all the porcine miRNAs were acquired from miRBase (http://www.mirbase.org/). miRNAs targeting GR were predicted with an online miRNA prediction tool [4]. Among all the predicted miRNAs, 4 miRNAs targeting GR were quantitated by real-time PCR. These four miRNAs are miR-124a, miR-142-3p, miR-30 and miR-204, the miRNAs binding sites on the 3′UTR of GR were shown in Fig. 1.

### 1.3 MicroRNA RT-PCR quantification

Total RNA was extracted from liver samples using the TRIzol reagent (Invitrogen) and subsequently purified with the RNase-Free DNase Kit (Promega) according to the manufacturer׳s instructions. For adding a poly-A tail to the end of each RNA transcript, the total RNA was treated with the Poly (A) Tailing Kit (Ambion, AM1350). The tailing reactions including 2 µg RNA samples (500 ng/ µl), 4 µl of 5× Escherichia coli poly (A) polymerase (E-PAP) buffer, 2 µl of 25 mM-MgCl_2_, 2 µl of 10 mM-ATP and 0.8 µl E-PAP (2 U/µl) adjusted to 20 µl with nuclease-free water. The 20 µl reactions were incubated for 1 h at 37 °C and held at 4 °C. Then, the sample was purified to remove any residual tailing reagents. Complementary DNA was synthesized from the tailed RNA using gene-specific primers with oligo-dT (a short sequence of deoxy-thymine nucleotides) adapters. RT reactions contained 2 µg poly-A-tailed miRNA, 1 µl oligo-dT adapter (1 µg/µl) and nuclease-free water. The 10 µl reactions were incubated at 70 °C for 5 min (RT1). The RT2 reactions including the entire RT1 reactions, mixed with 5 µl moloney murine leukemia virus reverse transcriptase (M-MLV) 5× buffer (250 mM, pH=8.3) Tris–HCl, 15 mM MgCl_2_, 50 mM dithiothreitol and 375 mM KCl, 1.25 µl of 10 mM-deoxyribonucleotide tripho-sphate, 1 µl M-MLV RNase (200 U/µl) and 0.5 µl RNase inhibitor (40 U/µl). The 25 µl reactions were incubated for 1 h at 42 °Cand then at 95 °C for 5 min. The 25 µl PCR mixture included 2 µl RT product, 2 µl primers, 8.5 µl sterile 3d H_2_O run on an Mx3000P instrument (Agilent Technologies) and analyzed using Mx3000P System SDS software (Stratagene). To evaluate miRNA expression, U6 small nuclear RNA (U6 snRNA) was used as a reference gene to normalize the expression of miRNAs. The Ct value is defined as the fractional cycle number at which the fluorescence passes the fixed threshold. The primer sequences used for miRNAs analysis are listed in Table 1.The fold change was calculated using the 2^-△△^Ct method. All experiments were carried out in triplicate. As shown in Table 2, among the 4 miRNAs targeting GR, miR-124a, which has been identified to target GR 3′UTR [2], [3], was significantly higher expression in the liver of betaine-exposed piglets, compared to that of control group.

## Figures and Tables

**Table 1:** Primers of miRNAs in this study.

Target genes	Sequences (5′ to 3′)
ssc-miR-124a	taaggcacgcggtgaatgcca
ssc-miR-142-3p	tgtagtgtttcctactttatgg
ssc-miR-30	tgtaaacatcctcgactggaag
ssc- miR-204	ttccctttgtcatcctatgcct
oligo dT adapter	tagagtgagtgtagcgagcacagaatt
aatacgactcactataggttttttttttttttttvn
Universal primer	tagagtgagtgtagcgagca
U6	ggcaaggatgacacgcaaat

**Table 2:** Expression of miRNAs predicted to target 3′UTR of GR in the liver of piglets.

Variables	Control	Betaine	*P*-value
ssc-miR-124a	1.00±0.11	1.52±0.12	<0.05
ssc-miR-142-3p	1.00±0.13	1.02±0.14	=0.68
ssc-miR-30	1.00±0.10	0.89±0.10	=0.24
ssc-miR-204	1.00±0.08	1.13±0.11	=0.19

Values are mean±SEM, *n*=8/group.

GR, glucocorticoid receptor.
